# The Therapeutic Influence of Heated Atmosphere

**Published:** 1846-01-01

**Authors:** 


*The Therapeutic influence of heated Atmosphere.*—Dr. Jules Guagot, of Paris, France, has published a work on the employment of heat as an external application in the treatment of ulcers, after surgical operations, in hysteria, rheumatism, &c., a notice of which we find in Ranking's half-yearly abstract of the medical sciences, vol. 1, No. 1. He applies the term "incubation" to indicate a bath local or general, in an atmosphere heated to ninety-six degrees Fahrenheit. He divides the remedy into 3 classes: 1st, *local*, when applied to ulcers, amputations, &c.; 2d, *diffuse*, when a considerable portion of the body is acted upon; 3d, *general*, when the whole body is exposed. He employs various kinds of apparatus, which, however, are not described in the article referred to.—According to the author of this practice, he has found it highly serviceable in ulcers of an inveterate character and long standing, in phlegmonous erysipelas, in eczema, white swelling, chronic rheumatism, &c.

